# Mild and non-persistent fasting plasma glucose elevation in the first trimester of pregnancy is not associated with increased risk of gestational diabetes mellitus and adverse pregnancy outcomes

**DOI:** 10.20945/2359-4292-2026-0077

**Published:** 2026-07-22

**Authors:** Natalia Gattass-Ferreira, Carlos Antonio Negrato, Ana Luiza de Mattos Telles, Gabriela Rogonsky da Costa, Claudia Saunders, Marcus Miranda de Oliveira, Melanie Rodacki, Lenita Zajdenverg

**Affiliations:** 1 Departamento de Clínica Médica, Universidade Federal do Rio de Janeiro, Rio de Janeiro, RJ, Brasil; 2 Faculdade de Medicina de Bauru, Universidade de São Paulo, Bauru, SP, Brasil; 3 Instituto de Nutrição Josué de Castro, Universidade Federal do Rio de Janeiro, Rio de Janeiro, RJ, Brasil; 4 Maternidade Escola da Universidade Federal do Rio de Janeiro, Universidade Federal do Rio de Janeiro, Rio de Janeiro, RJ, Brasil

**Keywords:** Gestational diabetes mellitus, early gestational diabetes mellitus, fasting plasma glucose, first trimester

## Abstract

**Objective:**

To evaluate pregnancy outcomes in women with early gestational diabetes mellitus (eGDM) diagnosed by mild elevation of fasting plasma glucose (FPG) in the first trimester.

**Subjects and methods:**

This prospective cohort study included 114 pregnant women with first trimester FPG <100 mg/dL (5.6 mmol/L). Women with FPG ≥92 mg/dL (5.1 mmol/L) and <100 mg/dL (5.6 mmol/L) were classified as having eGDM, and FPG was reassessed after approximately 3-4 weeks. Women whose repeated FPG was <92 mg/dL (5.1 mmol/L) were assigned to Group 1 (G1; n = 33) and did not receive eGDM treatment. These participants underwent an oral glucose tolerance test (OGTT) between 24-28 weeks of gestation and, depending on the results, initiated treatment as indicated. Women with eGDM and a second FPG 92-125 mg/dL (5.1-6.9 mmol/L) were assigned to Group 2 (G2; *n* = 31) and immediately started eGDM treatment. The control group (G3; *n* = 50) was composed by pregnant women with FPG <92 mg/dL (5.1 mmol/L) in the first trimester.

**Results:**

G1 and G3 had lower pre-pregnancy body mass index, less chronic hypertension, lower rates of GDM history, and lower multiparity compared to G2. There was no significant difference in GDM diagnosis by OGTT between G1 and G3 (27.3% vs. 20.0%, p = 0.594). G3 gained more weight (10.2 ± 7.6 kg, 7.5 ± 6.4 kg, and 13.5 ± 6.1 kg for G1, G2, and G3, respectively; p = 0.001). Other outcomes between G1 and G3 were similar.

**Conclusion:**

Women with eGDM diagnosed by mild, non-persistent FPG elevation in the first trimester had similar outcomes to those with normal early FPG.

## INTRODUCTION

Gestational diabetes mellitus (GDM), as defined by the World Health Organization (WHO), is a carbohydrate intolerance of variable severity first recognized during pregnancy and does not fulfill criteria for overt diabetes (^1^). The diagnosis is made using a 75-g oral glucose tolerance test (OGTT) between 24-28 weeks of gestation, with only one abnormal value required: fasting plasma glucose (FPG) ≥92 mg/dL (5.1 mmol/L) and <126 mg/dL (7.0 mmol/L); 1-hour ≥180 mg/dL (10.0 mmol/L); 2-hour ≥153 mg/dL (8.5 mmol/L) and <200 mg/dL (11.1 mmol/L). These cutoffs were established by the International Association of Diabetes and Pregnancy Study Groups (IADPSG) and the WHO, based on data from the Hyperglycemia and Adverse Pregnancy Outcomes (HAPO) study, which evaluated the relationship between maternal glucose and fetal outcomes (^1^-^3^). Although the HAPO study assessed women between 24-32 weeks of gestation and insulin resistance typically increases after late second trimester, FPG thresholds of ≥92 mg/dL (5.1 mmol/L) and <126 mg/dL (7.0 mmol/L) were extrapolated to all periods of gestation (^1^). The use of FPG ≥92 mg/dL (5.1 mmol/L) for early GDM diagnosis is not supported by first-trimester data, so neither FPG nor OGTT diagnoses in early pregnancy are evidence-based (^4^,^5^). In Brazil, the Ministry of Health recommends FPG at the first prenatal visit to detect overt diabetes. If FPG is ≥92 mg/dL (5.1 mmol/L) and <126 mg/dL (7.0 mmol/L), GDM is diagnosed without repeat FPG or OGTT (^6^).

To date, no studies have determined a clear cutoff for first-trimester FPG related to adverse maternal-fetal outcomes. The benefits of diagnosing and treating GDM before 24 weeks of gestation remain uncertain. One study reported that women diagnosed with GDM before 24 weeks had higher risks of pregnancy-induced hypertension, postpartum hemorrhage, postpartum glucose abnormalities, and their offspring had increased rates of prematurity, large-for-gestational-age, and neonatal intensive care unit (NICU) admission (^7^). A meta-analysis of 13 cohort studies found higher rates of perinatal mortality, neonatal hypoglycemia, and insulin use among women with early-onset GDM compared to late-onset GDM, as well as increased risk of NICU admission in developed countries (^8^). In a more recent trial, immediate treatment for GDM before 20 weeks in women with risk factors for hyperglycemia modestly reduced the incidence of a composite of adverse neonatal outcomes, particularly for those with higher glycemic values. The study also suggested that early treatment may increase risk of small-for-gestational-age (SGA) infants among women with OGTT results in the lower glycemic range (^9^).

Therefore, in addition to the uncertain benefits of early treatment, there is concern regarding overtreatment, undernutrition, and increased risk of SGA and NICU admission (^9^-^12^). In this study, we aimed to assess maternal-fetal outcomes in women with early GDM (eGDM) diagnosed by mild FPG elevation in the first trimester who did not undergo early intervention.

## SUBJECTS AND METHODS

### Study design

**Figure 1:**
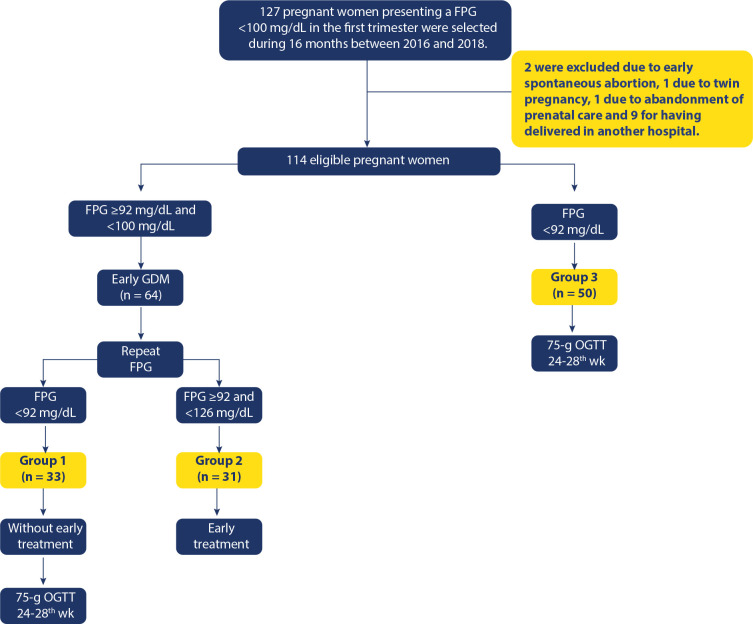
Study flow chart.

FPG: fasting plasma glucose; GDM: gestational diabetes mellitus; OGTT: oral glucose tolerance test.

This was a prospective cohort study. Pregnant women attending prenatal outpatient clinics at a maternity school hospital in Brazil over a 16-month period (2016-2018) with FPG <100 mg/dL (5.6 mmol/L) in the first trimester were included (**Figure 1**). Pregnant women with FPG ≥92 mg/dL (5.1 mmol/L) and <100 mg/dL (5.6 mmol/L) were classified as having eGDM. According to institutional protocol, FPG was repeated approximately 3-4 weeks (^13^).

Women with eGDM whose second FPG was <92 mg/dL (5.1 mmol/L) were assigned to group 1 (G1) and did not receive eGDM treatment. They underwent routine prenatal care and a 75-g OGTT between 24-28 weeks of gestation. Those with any abnormal OGTT values (FPG ≥92 mg/dL [5.1 mmol/L]; 1-hour ≥180 mg/dL [10.0 mmol/L]; 2-hour ≥153 mg/dL [8.5 mmol/L]) began medical nutrition therapy (^14^). Blood glucose monitoring devices were provided only for patients on insulin therapy. Diet-treated women were assessed every 2 weeks with FPG and 1-hour post-breakfast plasma glucose. Insulin was initiated for those with FPG ≥95 mg/dL (5.3 mmol/L) and/or 1-hour post-breakfast plasma glucose ≥140 mg/dL (7.8 mmol/L) persisting after at least two weeks on diet. Insulin doses were adjusted every 1-2 weeks by an endocrinologist based on self-monitoring of blood glucose (six times daily) aiming for fasting and preprandial glucose <95 mg/dL (5.3 mmol/L) and 1-hour postprandial glucose <140 mg/dL (7.8 mmol/L) (^15^).

Women in G1 with normal 75-g OGTT between 24-28 weeks of gestation were followed up to the end of pregnancy, in regular prenatal care until delivery with no specific GDM intervention. Women with eGDM and a second FPG ≥92 mg/dL (5.1 mmol/L) and <126 mg/dL (7.0 mmol/L) were assigned to group 2 (G2) and, for ethical reasons, immediately began GDM treatment with medical nutrition therapy (^14^). Insulin was added if glycemic control was not achieved on diet alone. The control group (G3) comprised pregnant women with first trimester FPG <92 mg/dL (5.1 mmol/L), selected by convenience from the same outpatient clinics during the inclusion period for G1 and G2. All underwent a 75-g OGTT between 24-28 weeks of gestation. Those with abnormal OGTT values were treated for GDM (^14^). Women who presented normal OGTT continued standard prenatal care until delivery.

The FPG cutoff value of ≥92 mg/dL (5.1 mmol/L) was chosen per IADPSG recommendations for GDM diagnosis (^2^). The upper limit of <100 mg/dL (5.6 mmol/L) was chosen because some experts suggest that FPG above this threshold is associated with an increased likelihood of developing GDM at 24-28 weeks and presenting unfavorable maternal and fetal outcomes, warranting dietary intervention (plus insulin if necessary) (^16^,^17^). Some studies have corroborated this information, demonstrating that over 50% of patients with first-trimester FPG >99 mg/dL (5.5 mmol/L) later develop GDM after 24 weeks gestation (^18^,^19^). Regarding the sample size, as the prevalence of mild first trimester FPG elevation in this population is unknown, all eligible women attending the clinic during the 16-month period (2016-2018) were considered for inclusion. Exclusion criteria were prior diabetes mellitus, current GDM treatment, multiple pregnancy, uncontrolled hyperthyroidism, or prior bariatric surgery. All eligible patients agreed to participate.

The research was approved by the local ethics committee (CAAE no. 58475616.3.0000.5275), and all participants provided informed consent.

### Data collection

At baseline, participants underwent interviews for clinical and anthropometric data. The International Physical Activity Questionnaire (IPAQ short form) (^20^,^21^) and a semi-quantitative food frequency questionnaire (^22^), both validated in Brazilian Portuguese, were administered. These were repeated between 24-28 weeks to detect changes in diet or activity potentially affecting outcomes. After delivery, maternal and fetal outcomes were obtained from medical records. Gestational age and expected delivery dates were established based on the last menstrual period. Ultrasonography (USG) dating was considered in cases where the date of the last menstruation was uncertain or differed by >5 days from the date obtained by USG performed between 6-13 weeks or >10 days from the date based on USG performed between 14-24 weeks.

### Outcomes

Maternal outcomes included GDM diagnosis by 75-g OGTT between 24-28 weeks, need for insulin therapy, gestational weight gain, gestational hypertensive disorders (preeclampsia: systolic blood pressure ≥140 mmHg or diastolic blood pressure ≥90 mmHg on two or more occasions after 20 weeks with proteinuria [≥1+ dipstick or ≥300 mg24/h]; gestational hypertension: if the criteria for elevated blood pressure were met but without proteinuria; eclampsia: presence of seizures in preeclamptic patients), and cesarean section.

Perinatal outcomes included prematurity (delivery <37 weeks), APGAR <7 at 1 or 5 minutes, large-for-gestational-age (birth weight >90th percentile for sex and gestational age), small-for-gestational-age (birth weight <10th percentile), macrosomia (birth weight ≥4000 g), neonatal hypoglycemia (glucose <36 mg/dL [2.0 mmol/L] in the first 24 hours), shoulder dystocia or birth injury, NICU admission, hyperbilirubinemia (requiring phototherapy), polyhydramnios, and intrauterine fetal or neonatal death.

### Statistical analysis

Data were analyzed using SPSS (v. 24.0, IBM Corporation, USA). Means and standard deviations or medians with ranges are reported for continuous variables; counts and percentages for categorical variables. Categorical variables were compared with chi-square or Fisher’s exact test (if expected frequency <5). Normality of continuous variables was assessed using the Shapiro-Wilk test. Parametric variables were evaluated with a Student’s t-test or analysis of variance (ANOVA), and nonparametric variables were evaluated with the Mann-Whitney U or Kruskal-Wallis test. Binary logistic regression (restricted to G1 and G2) was performed to assess whether higher NICU admission was associated with eGDM with early treatment (G2) or with other factors. Model variables were those with significant univariate differences (*p* < 0.05) between G1 and G2: pre-pregnancy body mass index (BMI), nulliparity, chronic hypertension, and lower carbohydrate intake at 24-28 weeks. Multiple linear regression (restricted to G1 and G2) was used to assess whether lower gestational age at birth was related to eGDM with early treatment (G2) or to other factors. Two models were constructed: the first included variables with significant univariate differences (*p* < 0.05) between G1 and G2; the second added insulin use (since insulin can influence delivery timing). Forward selection was used. Statistical significance was defined as *p* <0.05.

## RESULTS

A total of 127 pregnant women were recruited. Of these, two were excluded due to spontaneous abortion immediately after the first FPG collection, one due to twin pregnancy, one owing to discontinuation of prenatal care at the Institution, and nine because they delivered in another hospital. At the end, 114 pregnant women were eligible for analysis: 33 in G1, 31 in G2, and 50 in G3 (**Figure 1**).

Baseline characteristics are presented in **Table 1**. G2 exhibited a higher pre-pregnancy BMI (30.7 ± 6.0, 26.8 ± 5.5, and 27.5 ± 5.6 kg/m^2^ [*p* = 0.017], for G2, G1, and G3, respectively), a greater prevalence of chronic hypertension (48.4, 21.2, and 18.0% [*p* = 0.008], for G2, G1, and G3, respectively) and a higher frequency of personal history of GDM (23.1, 4.8, and 2.8% [*p* = 0.023], for G2, G1, and G3, respectively) than the other groups, along with a lower prevalence of nulliparous women compared to G1 (29.0% vs. 54.5% [*p* = 0.047]). G1 and G3 had similar baseline characteristics.

**Table 1:** Baseline maternal characteristics

Maternal characteristics (n = 114)	G1 (n = 33)	G2 (n = 31)	G3 (n = 50)	*p* ^^*^^	*p* ^†^	*p* ^‡^	*p* ^††^
n	33 (28.9%)	31 (27.2%)	50 (43.9%)				
FPG 1 (mg/dL) Gestational age (week) Fasting duration (hour)	94.0 (92-99) 8.9 (5.1-14.3) 10.5 (8-14)	95.0 (92-99) 9.4 (5.4-14.7) 11.0 (8-13)	83.0 (70-91) 9.6 (5-17.6) 12.0 (8-13)	0.000 0.444 0.244	0.085 0.619 0.781	0.000 0.180 0.196	0.000 0.610 0.145
FPG 2 (mg/dL) Gestational age (week) Fasting duration (hour)	86.0 (67-91) 13.1 (8.4-27.1) 10.0 (8-12)	97.0 (92-128) 12.7 (8.3-21.6) 10.0 (8-12)	-- -- --	0.000 0.519 0.926	0.000 0.519 0.926	-- -- --	-- -- --
Age (year)	27.7 ± 6.3	30.4 ± 6.4	29.4 ± 6.6	0.229	0.088	0.251	0.474
Pre-pregnancy BMI (kg/m^2^)	26.8 ± 5.5	30.7 ±6.0	27.5 ± 5.6	0.017	0.010	0.599	0.018
Non-White (n, %)	14 (42.4)	16 (51.6)	32 (64.0)	0.159	0.617	0.072	0.353
School attendance (n, %) ≤ 8 years 9-11 years ≥ 12 years	5 (15.2) 19 (57.6) 9 (27.3)	3 (9.7) 20 (64.5) 8 (25.8)	6 (12.0) 31 (62.0) 13 (26.0)	0.821 0.856 1.000	0.709 0.616 1.000	0.747 0.819 1.000	1.000 1.000 1.000
Family income (Brazilian minimum wage)	1.85 (0.26-8.80)	2.05 (0.53-5.56)	1.76 (0.35-0.56)	0.825	0.638	0.783	0.583
Nullipara, n (%)	18 (54.5)	9 (29.0)	22 (44.0)	0.127	0.047	0.377	0.241
Planned pregnancy (n, %)	11 (33.3)	14 (45.2)	18 (36.0)	0.623	0.443	0.819	0.486
History of GDM^§^ (n, %)	1 (4.8)	6 (23.1)	1 (2.8)	0.023	0.112	1.000	0.018
History of macrosomia^‖^ (n, %)	0	3 (13.6)	2 (7.1)	0.354	0.249	0.526	0.643
Family history of DM^¶^ (n, %)	13 (39.4)	11 (35.5)	12 (24.0)	0.302	0.800	0.150	0.315
Chronic hypertension^#^ (n, %)	7 (21.2)	15 (48.4)	9 (18.0)	0.008	0.035	0.780	0.006
History of PCOS^^**^^ (n, %)	8 (24.2)	3 (9.7)	8 (16.0)	0.294	0.186	0.401	0.518

BMI: Body mass index; DM: diabetes mellitus; FPG: fasting plasma glucose; GDM: gestational diabetes mellitus; G1: early gestational diabetes mellitus without early treatment; G2: early gestational diabetes mellitus with early treatment; G3: control group; PCOS: polycystic ovaries syndrome.

*Comparison between G1, G2 and G3.

^†^Comparison between G1 and G2.

^‡^Comparison between G1 and G3.

^††^Comparison between G2 and G3.

^§^Data were collected only in women with previous gestation. G1 (n = 20), G2 (n = 26), G3 (n = 36).

^‖^G1 (n = 16), G2 (n = 22), G3 (n = 28).

^¶^Parents or siblings.

^#^Hypertension diagnosed before pregnancy or before 20 weeks of gestation.

**Polycystic ovaries syndrome (self-declared).

Total daily caloric intake and macronutrient distribution at baseline and at 24-28 weeks’ of gestation are summarized in **Table 2**. Although overall caloric intake was similar across groups, women with early normal FPG (G3) had a higher percentage of lipid intake than the other groups at baseline (23.9 ± 5.0%, 24.0 ± 4.3%, and 26.7 ± 6.1% [*p* = 0.028], for G1, G2, and G3, respectively) and at 24-28 weeks (22.7 ± 3.8%, 24.2 ± 4.5%, and 26.7 ± 6.6% [*p* = 0.008], for G1, G2, and G3, respectively). At baseline, G2 consumed more carbohydrates than G3 (57.8% [41.6-76.0] vs. 51.4% [23.8-74.4] [*p* = 0.038], for G2 and G3, respectively), although this difference was not observed at 24-28 weeks. G1 and G2 had similar dietary patterns at baseline; however, at 24-28 weeks, G2 consumed less carbohydrate than G1 (54.8 ± 6.7% vs. 51.0 ± 4.9% [*p* = 0.025], respectively). Data on physical activity are described in **Table 2**. At baseline, G1 had a higher percentage of active patients than G3 (57.6% vs. 34% [*p* = 0.043], for G1 and G3, respectively). By the end of the second trimester, physical activity levels were similar across all groups.

**Table 2:** Food consumption and level of physical activity at baseline and at 24-28 weeks of gestation

Variables	G1 (n = 33)	G2 (n = 31)	G3 (n = 50)	p^*^	p^†^	p^‡^	p^††^
Food consumption baseline							
Daily calorie intake (kcal)	1871.5 (224.7-6071.2)	1907.3 (1109.1-4082.0)	1977.4 (605.1-5950.7)	0.864	0.624	0.845	0.669
Protein, %	18.9 (9.9-46.0)	18.2 (8.7-38.2)	20.5 (11.8-37.9)	0.414	0.489	0.533	0.193
Lipids, % CHO, %	23.9 ± 5.0 57.3 (31.8-65.7)	24.0 ± 4.3 57.8 (41.6-76.0)	26.7 ± 6.1 51.4 (23.8-74.4)	0.028 0.067	0.949 0.888	0.032 0.073	0.034 0.038
Food consumption 24-28 weeks^§^							
Daily calorie intake (kcal)	2033.70 (992.0-4454.7)	1954.40 (1281.4-3706.3)	1861.61 (1207.0-3278.6)	0.847	0.711	0.453	0.790
Protein, %	22.1 (13.7-34.3)	24.0 (15.3-35.3)	20.2 (10.7-39.1)	0.137	0.141	0.953	0.203
Lipids, %	22.7 ± 3.8	24.2 ± 4.5	26.7 ± 6.6	0.008	0.227	0.003	0.056
CHO, %	54.8 ± 6.7	51.0 ± 4.9	50.9 ± 9.5	0.081	0.025	0.056	0.783
Level of physical activity baseline							
Very active	0	1 (3.2%)	2 (4.0%)	0.619	0.484	0.515	1.000
Active	19 (57.6%)	17 (54.8%)	17 (34.0%)	0.062	1.000	0.043	0.063
Irregularly active A	8 (24.2%)	3 (9.7%)	13 (26.0%)	0.181	0.186	1.000	0.147
Irregularly active B	2 (6.1%)	7 (22.6%)	8 (16.0%)	0.183	0.078	0.302	0.763
Sedentary	4 (12.1%)	3 (9.7%)	10 (20.0%)	0.447	1.000	0.389	0.351
Level of physical activity 24-28 weeks^‖^							
Very active	1 (3.7%)	1 (3.6%)	0	0.326	1.000	0.397	0.406
Active	15 (55.6%)	15 (53.6%)	17 (41.5%)	0.467	1.000	0.323	0.339
Irregularly active A	3 (11.1%)	6 (21.4%)	9 (21.9%)	0.511	0.469	0.338	1.000
Irregularly active B	4 (14.8%)	5 (17.9%)	12 (29.3%)	0.362	1.000	0.245	0.395
Sedentary	4 (14.8%)	1 (3.6%)	3 (7.3%)	0.285	0.193	0.423	0.641

CHO: Carbohydrate; G1: early gestational diabetes mellitus without early treatment; G2: early gestational diabetes with early treatment; G3: control group.

Level of physical activity by IPAQ (21): Very active-any one of the following criteria: ≥5 days of vigorous activity of at least 30 minutes/day; or ≥3 days of vigorous activity of at least 20 minutes/day plus ≥5 days of moderate-intensity activity or walking of at least 30 minutes/day. Active-any one of the following criteria: ≥3 days of vigorous activity of at least 20 minutes/day; or ≥5 days of moderate-intensity activity or walking of at least 30 minutes/day; or ≥5 days and at least 150 minutes/week of any combination of walking, moderate-intensity or vigorous intensity activities. Irregularly active A-any of the following criteria regarding frequency or duration of activities: ≥5 days/week; or at least 150 minutes/week. Irregularly active B-those individuals who do not meet criteria regarding frequency or duration of activities. Sedentary-those individuals who did not perform physical activity for at least 10 continuous minutes/week.

* Comparison between G1, G2 and G3.

† Comparison between G1 and G2.

‡ Comparison between G1 and G3.

†† Comparison between G2 and G3.

§ G1 (n=28), G2 (n=28), G3 (n=41).

‖ G1 (n=27), G2 (n=28), G3 (n=41).

Regarding maternal outcomes are detailed in **Table 3**. FPG, 1h-PG, and 2h-PG during the 75-g OGTT at 24-28 weeks were similar in G1 and G3. The incidence of GDM diagnosed by OGTT at 24-28 weeks was also similar between G1 and G3 (27.3% vs. 20.0% [*p* = 0.594]). Among women with GDM, a higher percentage of those in G2 (early intervention) required insulin to achieve glycemic control compared to those in G1 and G3 (51.6, 22.2, and 10.0% [*p* = 0.036], for G2, G1, and G3, respectively).

**Table 3:** Maternal and newborns’ outcomes

Variables	G1 (n = 33)	G2 (n = 31)	G3 (n = 50)	*p* ^*^	*p* ^†^	*p* ^‡^	*p* ^††^
75-g OGTT (mg/dL) FPG 1-h PG 2-h PG	84.5 ± 8.1 127 (77-255) 109.5 ±21.8	- - -	82.5 ± 8.6 127 (64-197) 110.2 ± 24.5	0.290 0.366 0.897	- - -	0.290 0.366 0.897	- - -
GA at OGTT (week)	26.9 (23.4-35.7)	-	26.3 (21.7-34.6)	0.156	-	0.156	-
GDM (n, %)	9 (27.3)^§^	31 (100)^‖^	10 (20.0)^§^	-	-	0.594	-
GDM treatment Insulin (n, %) GA at the start of insulin (week) Insulin dose (units/kg/day) Basal insulin (n, %) Basal-bolus insulin (n, %)	2 (22.2) 32.0 ± 0.8 0.4 ± 0.3 2 (100) 0	16 (51.6) 24.3 ± 7.1 0.6 ± 0.3 7 (43.7) 9 (56.3)	1 (10.0) 32.3 ± . 0.4 ± . 0 1 (100)	0.036 0.237 0.592 0.334 0.334	0.149 0.161 0.427 0.471 0.471	0.582 0.821 0.940 0.333 0.333	0.028 0.299 0.487 1.000 1.000
Gestational weight gain (kg)	10.2 ± 7.6	7.5 ± 6.4	13.5 ± 6.1	0.001	0.132	0.031	0.000
Gestational weight gain (n, %) Bellow recommended^¶^ On target^¶^ Above the target^¶^	7 (21.2) 16 (48.5) 10 (30.3)	11 (35.5) 13 (41.9) 7 (23.6)	6 (12.0) 16 (32.0) 28 (56.0)	0.048 0.307 0.005	0.269 0.625 0.577	0.356 0.168 0.026	0.023 0.475 0.005
Gestational hypertension (n, %) Preeclampsia (n, %) Eclampsia (n, %)	0 5 (15.1) 0	0 9 (29.0) 0	1 (2.0) 7 (14) 0	1.000 0.208 -	- 0.232 -	1.000 1.000 -	1.000 0.150 -
Cesarean delivery (n, %) Primary Repeated	16 (48.5) 9 (27.3) 7 (21.2)	21 (67.7) 8 (25.8) 13 (41.9)	29 (58.0) 13 (26.0) 16 (32.0)	0.314 1.000 0.198	0.137 1.000 0.106	0.500 1.000 0.325	0.482 1.000 0.475
GA at delivery (week)	38.7 (22.7-41.3)	38.0 (24.7-40.7)	39.1 (22.1-41.3)	0.011	0.019	0.777	0.008
Prematurity (n, %)	2 (6.1)	5 (16.1)	8 (16.0)	0.383	0.250	0.302	1.000
Male sex (n, %)	16 (48.5)	13 (43.3)	23 (46.0)	0.970	0.801	1.000	1.000
1-min Apgar <7 (n, %)	3 (9.7)	1 (3.3)	2 (4.2)	0.564	0.612	0.376	1.000
5-min Apgar <7 (n, %)	1 (3.2)	1 (3.3)	0	0.311	1.000	0.392	0.385
Birth weight (g)	3315.0 (575-4145)	3185.0 (630-4315)	3240.0 (500-4630)	0.725	0.440	0.795	0.662
Large-for-gestational-age (n, %)	6 (18.7)	5 (16.1)	5 (10.4)	0.517	1.000	0.333	0.502
Small-for-gestational-age (n, %)	3 (9.4)	1 (3.2)	3 (6.2)	0.709	0.613	0.679	1.000
Macrosomia (n, %)	1 (3.1)	3 (9.7)	3 (6.0)	0.551	0.355	1.000	0.670
Neonatal hypoglycemia (n, %)	1 (3.2)	2 (6.4)	0	0.175	1.000	0.392	0.151
Shoulder dystocia or birth injury (n, %)	2 (6.4)	1 (3.2)	1 (2.1)	0.812	1.000	0.558	1.000
NICU (n, %)	4 (12.9)	13 (41.9)	9 (18.7)	0.014	0.021	0.552	0.039
Hyperbilirubinemia (n, %)	3 (9.7)	6(19.3)	3 (6.2)	0.195	0.473	0.674	0.143
Polyhydramnios (n, %)	0	2 (6.5)	0	0.072	0.231	-	0.144
Fetal death (n, %)	2 (6.1)	0	1 (2.0)	0.461	0.493	0.560	1.000
Early neonatal death (n, %)	0	0	0	-	-	-	-
Abortion (n, %)	0	0	1 (2.0)	1.000	-	1.000	1.000

FPG: fasting plasma glucose; GA: gestational age; GDM: gestational diabetes mellitus; G1: early GDM without early treatment; G2: early GDM with early treatment; G3: control group; NICU: neonatal intensive care unit; OGTT: oral glucose tolerance test; PG: plasma glucose.

* Comparison between G1, G2 and G3.

† Comparison between G1 and G2.

‡ Comparison between G1 and G3.

†† comparison between G2 and G3.

§ Diagnosis by OGTT between 24-28 weeks’ gestation.

‖ Early diagnosis by 2 altered fasting plasma glucose levels.

¶ Institute of Medicine recommendation (^23^,^24^).

The G3 gained more weight than the other groups (10.2 ± 7.6, 7.5 ± 6.4, and 13.5 ± 6.1 kg [*p* = 0.001], for G1, G2, and G3, respectively), with more than half of the women in G3 exceeding the weight gain recommended by the Institute of Medicine (IOM) (30.3, 23.6, and 56.0% [*p* = 0.005], for G1, G2, and G3, respectively) (^23^,^24^). No significant difference was observed in weight gain between G1 and G2.

Rates of gestational hypertensive disorders and primary cesarean section were similar across all groups. Fetal and neonatal outcomes are listed in **Table 3**. G2 had a lower median gestational age at delivery (38.7 weeks [22.7-41.3 weeks], 38.0 weeks [24.7-40.7 weeks], and 39.1 weeks [22.1-41.3 weeks] [*p* = 0.011], for G1, G2, and G3, respectively) and a higher rate of NICU admission (12.9, 41.9, and 18.7% [*p* = 0.014], for G1, G2, and G3, respectively) than the other groups, but no difference was observed in the rate of prematurity. G1 presented similar fetal and neonatal outcomes to G3. Multivariate analysis (using data from G1 and G2 only) indicated that eGDM treatment (G2), pre-pregnancy BMI, nulliparity, chronic hypertension, and lower carbohydrate intake at 24-28 weeks were not predictors for gestational age at delivery or NICU admission. In model II, insulin use was associated with gestational age at delivery (standardized beta -0.397 [*p* = 0.018]) (**Supplemental Table S1**).

## DISCUSSION

Our findings revealed that women with eGDM (diagnosed by mild, non-persistent FPG elevation in the first trimester) and fewer risk factors (e.g., obesity, multiparity, chronic hypertension) had outcomes comparable to those with early normal FPG, despite not receiving early intervention. To our knowledge, this is the first study to make such a comparison.

The group with eGDM that did not undergo early intervention showed similar fasting, 1-hour, and 2-hour plasma glucose values on the OGTT at 24-28 weeks to those with normal early FPG. The proportion diagnosed with GDM by OGTT at 24-28 weeks was also equivalent. Less than a third of G1 was diagnosed with GDM at 24-28 weeks and underwent intervention from then on, without worse outcomes. More than two-thirds of G1 would have undergone early intervention according to the current WHO GDM protocol, even in the absence of a GDM diagnosis by the present gold standard (75-g OGTT at 24-28 weeks). This is consistent with other studies showing that mild FPG elevations early in pregnancy (≥92 mg/dL [5.1 mmol/L] and <100 mg/dL [5.6 mmol/L]) are frequently not confirmed between 24-28 weeks, and fasting glucose generally declines throughout pregnancy across all pre-pregnancy BMI groups until the nineteenth week of gestation (^18^,^19^,^25^). Likewise, Cosson and cols. (^17^) showed that women with early fasting hyperglycemia (FPG between 92 mg/dL [5.1 mmol/L] and 125 mg/dL [6.9 mmol/L] before 22 weeks) and no risk factors are unlikely to develop GDM (^17^). In the TOBOGM study, GDM was newly diagnosed in 67% of women with eGDM who did not have early intervention; nevertheless, the control group in that study included women with risk factors, greater mean gestational age at screening (15.6 weeks), and higher initial FPG values (up to 109 mg/dL [6.0 mmol/L]) (^9^).

A question could be raised about the possibility that this first FPG of G1 would be slightly increased due to laboratory errors. Pre-analytical variables, such as the time between blood draw and centrifugation, are known to affect glucose measurement. Potter and cols. (^26^) noted that early centrifugation of 75-g OGTT samples can increase mean glucose readings and raise GDM diagnosis rates of 11.6-20.6%. In our study, all samples across groups were collected after overnight fasting in sodium fluoride tubes and analyzed under the same conditions and laboratory personnel.

It is also worth noting that although pregnant women in G1 did not undergo early intervention, these women gained less weight during pregnancy than those in G3, with most gaining weight below or within the threshold recommended by IOM (^23^,^24^). It is not clear if the lower weight gain favorably influenced the outcomes in G1, since it is not defined what is the appropriate weight gain cutoff to achieve better perinatal outcomes in pregnancies complicated or not by GDM (^27^,^28^). The rate of chronic hypertension in our study was higher compared with typical values reported in the pregnant population, which may be due to the fact that our maternity is a reference service for pregnant women with chronic hypertension.

Patients from G2 experienced lower gestational age at delivery and higher NICU admission rates than those in G1 or G3. While group allocation alone was not a predictor for NICU admission by multivariate analysis (examining G1 and G2 data only), the observed OR (2.65) suggests a trend potentially limited by sample size and number of events. Notably, the TOBOGM pilot study found an increased NICU admission rate among eGDM patients receiving early intervention (^12^). The lower gestational age at delivery in G2 likely reflects our institution’s protocol, which recommends interrupting pregnancy (by inducing vaginal delivery or cesarean section) in women with GDM in use of insulin at 38 weeks’ gestation. This was corroborated by the multivariate analysis (model II), in which only the use of insulin was associated with gestational age at birth. However, this difference did not translate into higher prematurity or adverse neonatal outcomes.

Moreover, previous studies have raised concerns regarding increased SGA incidence in eGDM cases given early treatment. In our study, there was no such increase in SGA in the early treatment group (^9^,^12^). Notably, G2 had a greater need for insulin compared to other groups, despite lower gestational weight gain, and similar findings have been reported elsewhere (^7^-^9^,^29^,^30^). This finding strengthens the position of some experts who defend early GDM diagnosis and treatment, positing that such patients present with more severe disease and may benefit from earlier intervention to reduce complications (^17^,^31^). However, there are concerns over potential overtreatment and resultant undernutrition (^8^,^10^-^12^,^32^). Our findings suggest that women with eGDM diagnosed by mild, non-persistent FPG elevation and with fewer risk factors had favorable outcomes even without early intervention, and their insulin use did not differ from control.

When we compared only the group that received early intervention (G2) with those with normal early FPG (G3), we noted that there were also no significant differences in most maternal-fetal outcomes. We speculate that this may be because G2 received early intervention protocol at our Institution. Another hypothesis is that even women with risk factors with mildly elevated FPG in the first trimester may evolve well throughout pregnancy, with or without early treatment. In the TOBOGM study, 33% of eGDM patients with risk factors who did not undergo early intervention no longer met criteria for GDM when assessed by the current gold standard (75-g OGTT at 24-28 weeks) (^9^). Future studies with larger sample sizes and dedicated designs should seek to address these questions.

Our study has some limitations, including the small number of patients, absence of formal sample size estimation, and the single-center, tertiary-care setting. For ethical reasons, we adhered to institutional protocols, initiating intervention for women with two early FPG readings ≥92 mg/dL (5.1 mmol/L). Nonetheless, this study’s strengths include the fact that it was conducted in a center where all participants received care from the same multidisciplinary team using a single protocol. Consequently, it was possible to perform a comparative analysis of various factors that could interfere in the evaluation of gestational outcomes.

In summary, this study found that women diagnosed with eGDM based on mild and non-persistent FPG elevation in the first trimester, and who did not receive early treatment, had outcomes similar to those with early normal FPG. These findings prompt reconsideration of glucose thresholds and the need for early intervention in pregnant women with fewer GDM risk factors. Larger, multicenter, well-controlled studies are warranted to evaluate the true benefit of early treatment for mild first-trimester FPG elevation.

## Figures and Tables

**Supplemental Table S1:** Multivariable analyses of predictors that characterized gestational age at delivery in early GDM groups and the need for admission in neonatal intensive care unit

Variables	NICU admission			Gestational age at delivery				
	OR (95% IC)	*p*		Model 1			Model 2^*^	
				Beta	*p*		Beta	*p*
G2	2.647 (0.64-10.93)	0.18		0.29	0.04		0.000	0.999
Pre-pregnancy BMI (kg/m^2^)	1.05 (0.91-1.22)	0.48		-0.08	0.62		-0.181	0.276
Nullipara	1.99 (0.35-11.33)	0.44		0.06	0.67		0.173	0.286
Chronic hypertension	1.09 (0.25-4.78)	0.91		-0.18	0.23		-0.170	0.296
Food consumption 24-28 week: CHO-%.	0.88 (0.76-1.02)	0.10		0.06	0.68		0.209	0.200
Use of insulin							-0.397	0.018

BMI: body mass index; CHO: carbohydrate; GDM: gestational diabetes mellitus; G2: early GDM with early treatment; NICU: neonatal intensive care unit.

* Model 2 included the use of insulin.

## Data Availability

datasets related to this article will be avail-able upon request to the corresponding author.
